# Ultrasonic Deposition of Carbon Nanotubes on Polycrystalline Cubic Boron Nitride Composites

**DOI:** 10.3390/ma14030516

**Published:** 2021-01-21

**Authors:** Manuela Pacella, Sina Saremi-Yarahmadi, Luciano Lamberti

**Affiliations:** 1 Wolfson School of Mechanical, Electrical and Manufacturing Engineering, Loughborough University, Loughborough, Leicestershire LE11 3TU, UK; 2 Department of Materials, Loughborough University, Loughborough, Leicestershire LE11 3TU, UK; S.Saremi@lboro.ac.uk; 3 Dipartimento di Meccanica, Matematica e Management, Politecnico di Bari, 70125 Bari, Italy; luciano.lamberti@poliba.it

**Keywords:** polycrystalline cubic boron nitride, ultrasonic bonding, carbon nanotubes, bonding of dissimilar materials, surface processing, micro-hardness

## Abstract

Polycrystalline cubic boron nitride (PcBN) are super-hard materials with high hardness and excellent abrasive resistance, widely used in cutting tools for precision machining of automotive and aerospace parts; however, their brittle properties make them prone to premature failure. Coatings are often applied to PcBN to extend their range of applicability and durability. Conventional coating methods are limited to the thickness range of a few hundred nanometres, poor adhesion to the substrate, and limited stability under ambient conditions. To further the properties of PcBN composites, in this paper, we explore the use of ultrasonic bonding to apply thick coatings (30–80 μm) on PcBN cutting tools. For the first time, a multi-walled carbon nanotube (MWCNT) powder is preplaced on a PcBN substrate to allow an unconventional coating technique to take place. The effects of ultrasonic bonding parameters on the change of mechanical properties of the coated tools are investigated through scanning electron microscopy (SEM), energy dispersive X-ray spectroscopy (EDX), micro-hardness analyses, and white light interferometry. The structure of the carbon nanotubes is investigated through transmission electron microscopy (pre coating) and cross-section of the bonded MWCNTs is studied via focused ion beam milling and SEM to evaluate the bonding between the multi-walled nanotubes. Optimum processing windows (i.e., bonding speed, energy, and pressure) are discovered for coating MWCNTs on PcBN. Focus ion beam milling analyses revealed a relationship between consolidation parameters and porosity of MW(pCNT) bonds. The proposed method paves the way for the novel design of functional coatings with attunable properties (i.e., thickness and hardness) and therefore improved productivity in the machining of aerospace and automotive parts.

## 1. Introduction

Polycrystalline cubic boron nitride (PcBN) composites are super-hard materials with high hardness and excellent abrasive resistance, used in place of diamond for machining of ferrous materials. However, tribo-oxidation and abrasion can occur during machining of hardened steel, leading to tool failure. Coatings are often applied to PcBN to extend their range of applicability by attuning their properties to the specific requirements of the machining task. Coatings on tools are crucial as they can act as a thermal barrier reducing the temperature of the tool-workpiece and retaining the heat within the deformation zones. Due to their low thermal conductivity and thermal stability, nanocomposite coatings provide thermal insulation and reduce the PcBN temperature in the cutting process, thereby reducing the coefficient of friction, the frictional energy (heat generation), and the chemical wear rate. Carbon nanotubes (CNT) are widely used in nanocomposite coatings in the automotive and electronics industry. Carbon nanotube has many beneficial properties, which include good tensile strength, good elastic properties, and excellent electrical and thermal conductivity [1]. It is worth noting that whilst excellent mechanical properties such as elastic modulus of >1 TPa are expected due to sp^2^ hybridisation of C-C bonds, factors such as chirality and diameter of the CNTs also impact these properties [2]. For example, average elastic modulus of multi-walled carbon nanotubes (MWCNTs) has been reported between 350 [3] and 1800 GPa [4] and tensile strength between 11 and 63 GPa. State-of-the-art research [1] demonstrated that the performance of PcBN cutting tool materials can be improved by adding CNTs to different coating formulations through physical vapour deposition (PVD) processes. Enhanced wear resistance was reported, and hardness and yield strength increased by a factor of 1.3. Further experiments investigated the effect of adding CNTs in iron-based binder in tools. The results showed that an addition of less than 0.1% concentration of CNTs increased hardness, strength, and Young’s Modulus, but led to a decrease in grain size. This was due to the binder recrystallisation process being hindered due to the nanotube particles at grain boundaries, which also prevented coating oxidation [5]. Coatings on PcBN tools are typically 2–3 μm in thickness, therefore they cannot compensate for a poor substrate with surface defects and nonhomogeneous surface roughness. Conventional coating methods (PVD or chemical vapour deposition, CVD) create a lubrication effect and reduce friction and temperatures at the cutting edge. Drawbacks of such approaches are the presence of droplets in the coating surface, which originate coating roughness, creating a deleterious effect on the lifetime stability, poor adhesion with the tool substrate [6] limiting thickness to a few hundred nanometres, and poor coating stability under ambient conditions.

In this context, the present paper aims at investigating the feasibility of manufacturing coatings with tunable architectures (i.e., thickness and mechanical properties) onto PcBN tools via ultrasonic bonding. Ultrasonic bonding is a hybrid sheet lamination manufacturing process used in key industries such as automotive and medical to join similar/dissimilar materials. This is a highly efficient, sustainable, and fast bonding method, especially when compared to other unconventional bonding processes (e.g., laser sintering or electron beam melting). It is faster than conventional adhesives, drying time is quick, and a more precise welding occurs. Furthermore, the lower temperature at which it operates can be beneficial as it has less impact on the mechanical properties [7]. Ultrasonic bonding uses high shear frequency mechanical vibration to generate a friction-like shear relative motion between the two sheets of material. Typically, the bonding mechanism can be dominated by solid-state bonding [8], local melting [9], and mechanical interlocking [10]. Process parameters, which have been shown to significantly affect the resulting bond, are ultrasonic frequency, vibration amplitude, clamping force, power, and energy. Ultrasonic frequency generated by the transducer is typically varied between 20 and 40 kHz. Vibration amplitude of the sonotrode is seen as an important parameter affecting the bonding quality as mechanical energy is transferred to the weld [7]. Previous studies on ultrasonic bonding of metals investigated the effect of bonding parameters on mechanical properties such as hardness, tensile strength, and fatigue behaviour of the bonded materials. Increasing the ultrasonic amplitude, for example, promoted a decrease in hardness of aluminium 1100 due to the dynamic recrystallisation in the aluminium sheets [7]. Hardness was affected by deformation-induced excess vacancy concentration generated due to the high strain rate dynamic deformation and material properties. Furthermore, the hardness distribution across the sheet varied because of cold working, making the centre weaker. For bonding occurring at lower weld energy, the sample was softer only near the sonotrode tip. Higher weld energy resulted in softer areas further away from the tip, producing a uniform hardness distribution. Tensile strength was measured under quasi static loading conditions which involved tensile shear, U-peel, and T-peel tests. The tensile strength varied from 1.35 kN for a 0.4 mm thick sheet (AA6061-T6) to 3.5 MPa for a 0.92 mm thick sheet (Al6111-T4). This was compared to weld energy; as it increases, the tensile strength also increases until it reaches a maximum, where it starts decreasing. Increasing weld energy increases the temperature, which causes metallurgical adhesion and mechanical interlocking across the weld. Higher temperature led to softening of the material [7].

Joining dissimilar materials is a difficult process due to the difference in mechanical and microstructural properties, which could lead to the formation of undesirable brittle intermetallic compounds. Research experiments have been carried out in bonding aluminium alloy 3003 and stainless-steel 304 through ultrasonic spot welding [11]. Parameters investigated included clamping pressures and energy levels to understand their effect on the microstructure, mechanical properties, and bonding quality. Different levels of bonding quality were defined in terms of extent of weld energy provided during the process. Weld energies between 125 and 150 J led to maximum bond strength. As the weld energy increased, the bond strength also increased. When looking at the microstructure, the original granular structure disappeared and a recrystallised region formed, which was attributed to the temperature rise as a result of the use of ultrasonic energy. Lap shear tensile test showed that the bonding produced using a low weld energy of 75 and 100 J failed at a small tensile load due to cold worked microstructure. When weld energy was increased above 150 to 200 J, an over weld formed, and the strength of the bond was decreased due to softening and thinning caused by the recrystallisation at the boundary. When characterising the hardness changes as a function of the weld energy, it was found that samples welded at 75 and 100 J showed higher hardness values. This was due to a large amount of cold working occurring at the boundary. Lower weld energy produced larger hardness values closer to the sonotrode as opposed to the regions further away [11]. A previous study on bonding dissimilar metals [12] investigated the effect of weld time on interface temperatures, demonstrating that using lower weld time (in the region of 0.5 s) might lead to voids due to the formation of less interface temperatures and smaller strain rate, while an increase of weld time (above 0.9 s) might cause a decrease of joint strength due to excessive plastic deformation and microcracks formation at the periphery of the weld zone.

In the need to increase the wear resistance of polycrystalline boron nitrides and enhance the microstructural and mechanical properties of these materials, the objectives of the proposed research are two-fold. Firstly, the ultrasonic bonding technique conventionally used to bond aluminium sheets is unconventionally applied here to coat boron nitrides with layers of MWCNTs of different thicknesses and properties. Secondly, an experimental study is proposed to investigate the effect of ultrasonic parameters on the mechanical, chemical, and topographical properties of the coated composites, showing promising enhancement of the mechanical properties of these materials.

## 2. Materials and Methods

### 2.1. Materials

**Figure 1:**
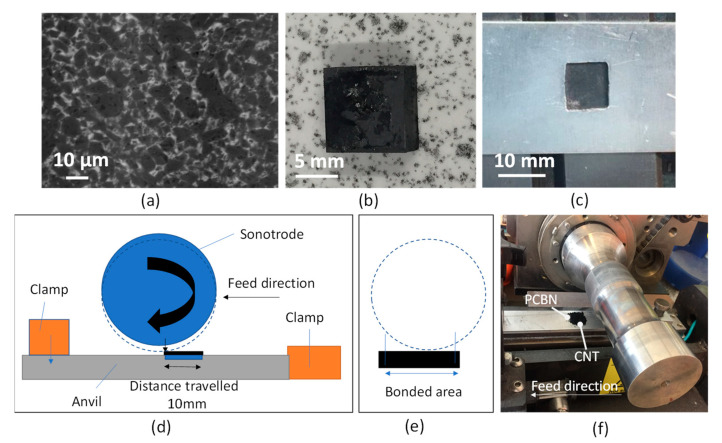
(**a**) Scanning electron microscope (SEM) micrograph of polycrystalline cubic boron nitride (PcBN) prior to coating, (**b**) PcBN square solid specimen for coating, and (**c**) tool-holder, (**d**) ultrasonic machine schematic, (**e**) area of bonding in cross-section, and (**f**) joining apparatus.

A commercially available solid PcBN material in the form of squares (10 × 10 × 5 mm^3^) shown in Figure 1b was selected for this study. This is a commercial grade with a high cBN content (wt% 90%) and an average cBN grain size of 15 µm and AlN and AlB2 as binder phases. Microstructural composition before coating is shown in Figure 1a.

MWCNTs with more than 98% purity (TNM3 grade, Chengdu Organic Chemicals Co. Ltd.) (Chengdu, China) were used to coat the PcBN tools. The specific surface area of the CNTs were >150 m^2^·g^−1^ with the tap density of 0.22 g·cm^−3^. The dimensions of the nanotubes range from 10 to 20 nm in diameter with typical lengths of 10–30 μm. These are a commercial grade from Chengdu Organic Chemicals Co Ltd. produced via the catalytic carbon vapour deposition (CCVD) process.

### 2.2. Bonding Method

A tool-holder was manufactured to keep the sample firmly in place during ultrasonic bonding: this was made of an aluminium plate long enough to be fixed in the conventional machine clamping system and an engraved square was prepared in the centre of the aluminium plate to hold the specimen (Figure 1c). An ultrasonic bonding machine (Alpha 2) was used for the experiments. The ultrasonic machine was connected to a filtered compressed air source. The operator interface panel allowed a change of process parameters: weld speed, jog speed, and horn power. The weld force was controlled by adjusting the applicable knob on the front pneumatics panel. The weld distance was set at 10 mm, as shown in Figure 1a. The sonotrode was moved in the feeding direction to allow precision in the identification and positioning of the bonding area. The bonding started when the ‘weld left’ and ‘weld right’ button were pressed. Prior to the bonding process, the weld area was cleared of any debris. The aluminium fixturing sheet was placed under the sonotrode and clamped using the supports on each side. The PcBN specimen was positioned in the fixture. A small ampoule (volume of 2000 mm^3^) was used to measure the volume of CNT powder to be preplaced on the substrate. This was done to ensure a homogeneous application of a layer of carbon nanotube powder of circa 200 μm based on sample volume calculations. The edges were aligned to ensure straight welding and the sonotrode was jogged to 50 mm from the edge of the clamp, then the sonotrode was dropped onto the material and rolled while feeding along the specimen’s length of 10 mm. A constant normal force and sonotrode oscillation frequency were applied during the process. High-frequency ultrasonic friction between the surfaces raised the temperature in contact causing the bonding to occur.

The experiment on PcBN focused on understanding the effect of welding parameters on the surface roughness, micro-hardness, and thickness and distribution of the coatings. The selected factors were welding force, welding speed, weld power, and weld time. These were selected at three levels (Table 1). This design of experiment (DOE) resulted in a Taguchi L7 orthogonal array. This set of 7 experiments was conducted 2 times for repeatability validation. The analysis of variance (ANOVA) was performed to evaluate the significance of interactions and main effects for the considered factors.

### 2.3. Material Characterisation Method

All test specimens were characterised in term of their micro-hardness, surface morphology, thickness of coating, and surface chemistry. The coating thickness and surface topography were measured using a high-resolution optical three-dimensional (3D) measurement system (Alicona InfiniteFocus) (Graz, Austria) while morphology and surface chemistry analyses were conducted using a scanning electron microscope (SEM) (XB1540 Carl Zeiss) (Oberkochen, Germany) equipped with an energy dispersive X-ray spectroscopy (EDX). Two-dimensional (2D) and 3D surface roughness measurements were taken prior/post-coating process using ISO 13565 (https://www.iso.org/obp/ui/#iso:std:iso:13565:-2:ed-1:v1:en) and ISO 25178 (https://www.iso.org/obp/ui/#iso:std:iso:25178:-2:ed-1:v1:en). A transmission electron microscope (TEM) (FEI Tecnai™ F20) (Hillsboro, OR, United States) was used to allow characterisation of the MWCNTs prior to coating.

Initial investigations of the coated samples via SEM allowed to identify suitable sites for milling of the coating’s cross-section at different bonding conditions to enable examinations at micro/nanometric resolution. An FEI Nova 600 Nanolab Dual Beam Focused Ion Beam (FIB) SEM was used to mill the cross-section of selected coating conditions up to a depth of 10 μm.

An initial deposition of a thin platinum layer (1 μm) was carried out in the site of milling in order to protect the entire exposed surface prior to FIB milling.

Hardness indentation tests were carried out using a Mitutoyo HM-102 Vickers Hardness Indenter (Kanagawa, Japan). The indenter tip used was a Vickers indenter (19BAA114) with an angle of 136°. All indentations were performed at room temperature (23 °C) in a controlled environment. For the indentation process, a load of 1 kg (9.807 N) was applied for a dwell time of 15 s. Due to the low magnitude of the load for PcBN materials, smaller indents were created during the micro-indentation hardness measurements. Indentations were made in five different locations for each specimen prior to and post-coating process.

## 3. Results and Discussion

The complete design of experiment used to investigate the effect of weld energy, amplitude, weld force, speed, and time on the mechanical properties of the achieved coating, coating uniformity, and thickness is reported in Table 2. Experimental investigations showed a strong correlation between quality of bonding and sonotrode’s amplitude of oscillation, weld force exerted from the sonotrode on the materials to be bonded, and the weld speed of the sonotrode.

### 3.1. Effect of Weld Energy and Pressure on Micro-Hardness and Coating Homogeneity

**Figure 2:**
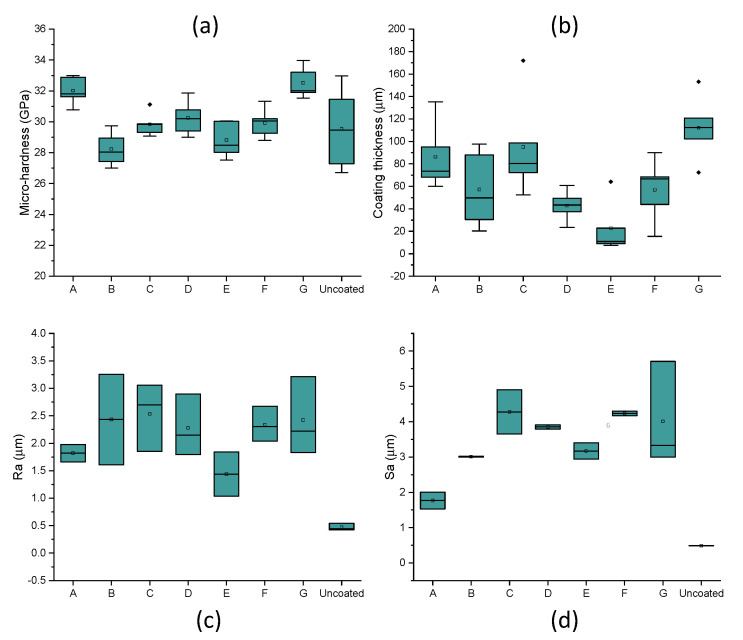
(**a**) Micro-hardness measurements for coated and uncoated samples, (**b**) coating thickness variation post-process, (**c**) boxplot of two-dimensional (2D) surface roughness (Ra) for coated and uncoated samples, (**d**) boxplot of 3D surface roughness (Sa) for coated and uncoated samples.

Analysis of variance (ANOVA) was conducted to evaluate the significance of main and interaction effects for all considered factors. The main aim was to identify the factors that enhance the mechanical properties (i.e., micro-hardness) and to functionalize the coating’s thickness. Table 3 shows the results only of significant interaction effects of DOE on micro-hardness for boxplots reported in Figure 2. For a confidence level of 95% (α = 0.05), the difference of the means is significant for all interaction effects (amplitude, weld speed, force, and power).

**Figure 3:**
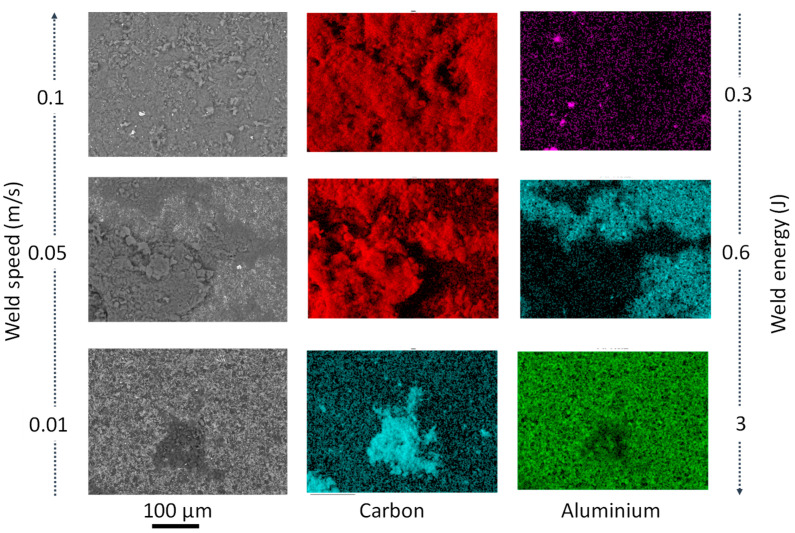
Schematic of the effect of weld speed and weld force variation on the elemental composition results for PcBN samples post-coating (from top to bottom, tests F, D, and E).

From the analysis of variance, the most influential process parameters on hardness and coating quality were weld energy and bonding pressure. As the weld energy and force increase, the hardness increased. This was due to the temperature increase (up to 800 K based on Matsuoka [13]) typical of ultrasonic welding processes, which caused metallurgical adhesion and mechanical interlocking across the weld, especially for bonding dissimilar materials. The effect of bonding energy is certainly one of the most critical factors in the conducted study. A change in bonding mechanism was evident at higher bonding energy (>0.6 J) and lower speeds (<0.05 m·s^−1^). In fact, when coating at slower speeds (<0.01 m·s^−1^), the MWCNT powder did not bond to the PcBN substrate (Figure 3). In bonding dissimilar materials, full interfacial contact and interdiffusion should occur. Typically, the voids present on the two contacting surfaces (coating and substrate) can be filled by surface diffusion or can be collapsed by creep [14], this phenomenon could occur before diffusion across the interface takes place [15]. It must be noted that diffusion is not essential to guarantee atomic-level bonds, however if it occurs it enables full bonding at the interface. During the bonding process the pressure plays an essential role in the attainment of full interfacial contact: a threshold pressure which enables the faces to be forced together exists for which no three-dimensional voids are present, however insufficient interdiffusion occurs. This might be the case for test E, where full interfacial contact did not occur and the coating only adhered to a small area of the sample, in opposition to test F, for which the coating deposited successfully. In fact, comparing conditions E and F, although the bonding energy was higher (3 versus 0.3 J) and it was applied for a longer time (1 versus 0.1 s), the impact of weld speed (0.01 versus 0.1 m·s^−1^) was significant in affecting the instantaneous pressure between the two contacting surfaces. It was expected that slower speeds and longer deposition time would facilitate diffusion, but those results might evidence that a full range of parameters should be considered for diffusion to be the dominant bonding mechanism. From the analysis of the elemental composition achieved via EDX, two main elements were found on all achieved coatings: carbon and aluminium. The first one expected as a dominant element of the CNTs and the latter being infiltration from the PcBN substrate. Elemental composition analyses in Figure 3 indicated a trend in increased porosity with the increase of energy, as evident from the patches of carbon in conditions D and E (Figure 3).

The 2D topographical data were extrapolated from the scanned profiles using ISO 13565. All 2D measurements for the coated samples resulted in higher roughness compared to the benchmark, with average Ra values in the range 2.4–2.5 μm for tests B, C, D, F, and G, 1.68 μm for test A, and significantly lower for test E (1.4 μm); however, all were significantly higher than the uncoated samples which had an average Ra of 0.5 μm (Figure 2c). ISO 25178 3D was used to derive the 3D surface roughness parameters Sa from the Abbot-Firestone Curve, which is indicative of the coating homogeneity. All 3D measurements for the coated samples resulted in higher Sa values compared to the benchmark, with average Sa values in the range 3–4.2 μm for tests B to G, and significantly lower for test A (1.75 μm); however, all were significantly higher than the uncoated samples, which had an average Sa of 0.5 μm (Figure 2d). The effect of amplitude on the 2D and 3D surface roughness parameters was also observed. From the topographical analysis, test C (12 μm) showed an average surface roughness, Ra, of 1.85 μm, and the Spk mean value derived from the Abbot-Firestone curves was 6.084 μm. Test D (21 μm) revealed lower 2D (Ra = 1.795 μm) and 3D (4.862 μm) parameters. As amplitude increases, the value of Spk decreases, which shows the wear resistance being greater at a higher amplitude.

Weld energy was varied to understand its effect on the hardness of the coated samples. Micro-hardness values for the uncoated PCBN sample ranged from 27.41 to 31.58 GPa. For bonding speeds above 0.05 m·s^−1^ and bonding energies below 0.6 J (condition A and F in Table 2), micro-indentation tests revealed an improvement of average hardness from 29.5 to 32 GPa. Test A (weld energy of 0.6 J) showed the largest hardness values with the average of 31.82 GPa. Using lower bonding pressures (5 MPa in Test B) resulted in softening up to 28.4 GPa. Higher bonding pressures (30 MPa) and high weld energies (1.2 J) in Test D resulted in an increase of average hardness up to 30.16 GPa. Similar values (30.02 GPa) were reported for Test F, in which the bonding pressure was similar to test D (circa 30 MPa), but the weld energy was lower (0.3 J). Overall, 5 coating conditions showed micro-hardness values in the range of the uncoated PCBN values, however smaller scatters in the boxplot were seen (Figure 2a), signifying more uniformity of mechanical properties post-process. Comparing Tests A and D, it is evident that bonding pressure has a higher impact than energy on hardness. Tests A and G revealed an average value of hardness above 32 GPa, well above the average value in the uncoated sample (29 GPa). The maximum bonding force (600 N) and bonding pressure (60 MPa) would have impacted the frictional heat generated during the bonding process, causing oxidation, breaking down of organic membranes, and dispersion by the vibration energy. When the surface roughness of the contacting surfaces differs, the welding pressure acting on each contact point may vary, and therefore the deformation [13]. After contact, bonding occurs through plastic and elastic deformation. There is a rise in temperature at the boundary due to vibration, which promotes atoms’ bonding. Matsuoka [13] reported that the temperature generated by heat-regenerative power in ultrasonic bonding (at pressures of 8.6 MPa and bonding duration of 0.7 s) reaches 793 K. Previous studies [8,16,17] also reported that there is a direct link between amplitude and measured temperature in ultrasonic bonding, and that for amplitudes of oscillation above 25 μm, the peak temperature was measured under 523 K, and for amplitudes of oscillation below 25 μm, the temperature was reported to be less than 373 K. Based on previous research [8,13,14,15], the temperature in conditions A and G could be in the range 450–793 K, with a likelihood of 95% for the achieved temperature to be above the required one for plastic deformation in 90% cBN materials, which is reported to be 473 K [18], causing plastic deformation of the cBN grains, strain hardening, and therefore, providing a strengthening effect.

**Figure 4:**
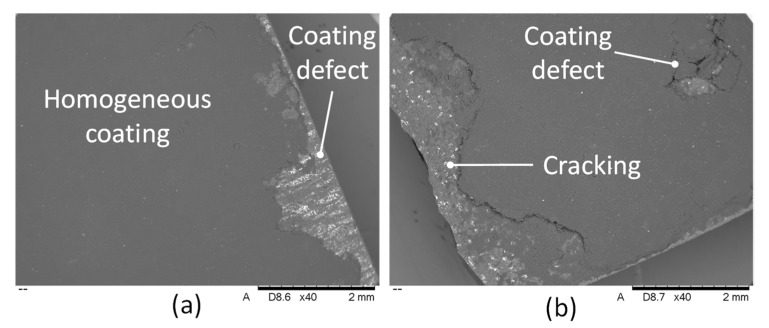
SEM images detailing the edge of the sample of Test A: (**a**) undeposited coating, (**b**) coating cracking.

Langenecker [19] and later Gunduz et al. [20] proved that diffusion in ultrasonic bonding can be enhanced by an increase of dislocation density due to plastic deformation at very high strain rates. This validates the hypothesis that diffusion due to plastic deformation of the cBN grains might have occurred in bonding at conditions A and G. The scatter in the micro-hardness boxplots (Figure 2a) for all coated samples resulted to be smaller than for the uncoated sample. This is because the hardness of cBN (single crystal) is anisotropic and it ranges from a minimum of 29.89 GPa in the (110) direction on the (001) plane to 43.12 GPa in the (100) direction on the (001) plane [21]. However, the measurements reported in this paper for the uncoated samples are in line with data from Reference [21]. Weld energy was varied for Tests A, B, D, and F. From the optical microscopy analysis of Test A (0.6 J), it was observed that the carbon nanotube coating was applied in most areas of the PcBN sample (Figure 4a). The sonotrode was dropped onto the sample at the edge and rolled for 10 mm before moving the sonotrode away to remove the force applied. However, due to the large force applied (600 N) and due to dragging of the sonotrode before lifting, the PcBN sample cracked on one side due to its brittle properties. Also, the carbon nanotube coating did not bond strongly near the edges, which may be likely due to the pressure applied by the sonotrode and which was suddenly removed. Yang et al. [22] demonstrated that weld pressure favours plastic flow during the bonding process only in combination with ultrasonic oscillations, which are key to produce stresses at the interface. This is in line with what was observed while bonding at conditions A and G, where the combination of high pressure and high amplitude facilitated plastic flow at the interface, evidenced by the highest increase of hardness.

Analysis of variance (ANOVA) was conducted to evaluate the significance of main and interaction effects for all considered factors on coating’s thickness. Table 4 shows the results only of significant interaction effects of DOE on thickness for boxplots shown in Figure 2. For a confidence level of 95% (α = 0.05), the difference of the means is significant only for the interaction effects shown in Table 4, namely weld energy, speed, power, and force.

**Figure 5:**
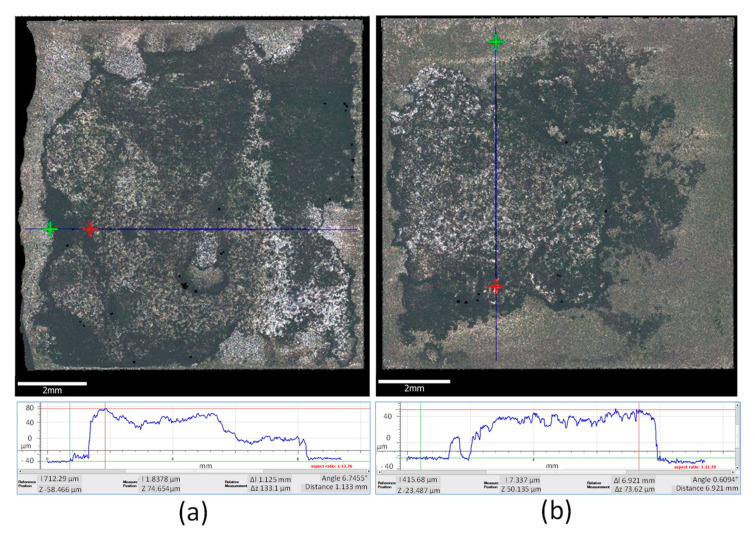
Focus variation microscopic thickness measurement of: (**a**) Test A, (**b**) Test B.

The coating thickness was measured across 5 areas of each PcBN sample, Test A had an average thickness of 86.45 μm. The centre of the sample had the thickest layer of coating (135.34 μm). From the optical image of Test B (0.5 J), the MWCNTs were only present at the centre, leaving an edge of 0.5–2 mm width uncoated (Figure 5b). Comparing Test B to the coating achieved using the highest energy (Figure 5a), less deposition areal coverage is achieved, confirming that the weld energy strongly affects the mechanical interlocking and diffusion processes. The coating thickness for Test B was measured at an average of 47.09 μm. The strong significance was also evident from the ANOVA results shown in Table 4 for conditions C and E.

**Figure 6:**
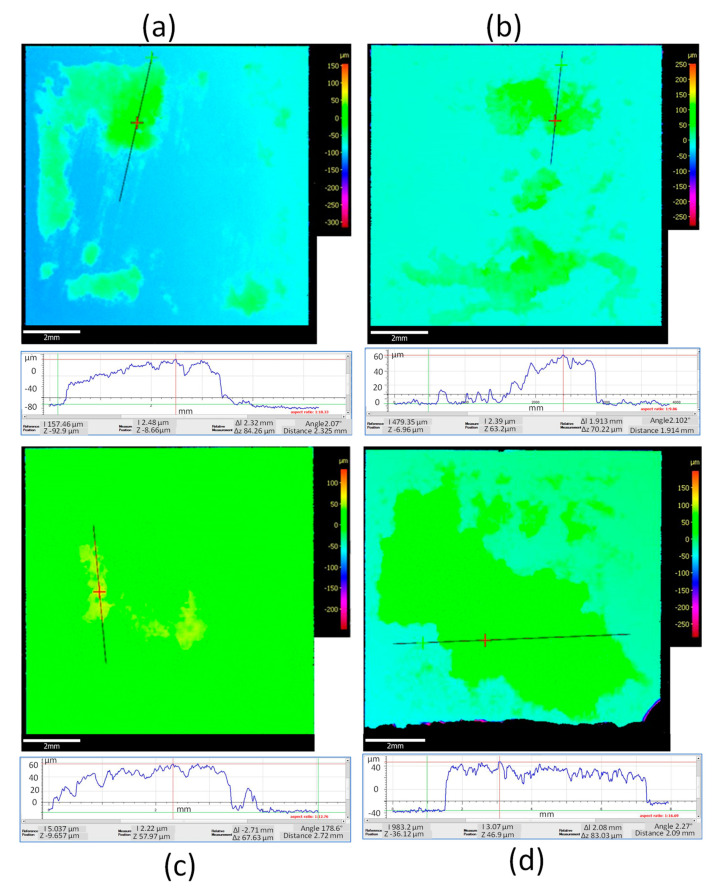
3D mapping of the thickness of the coated samples: (**a**) Test C, (**b**) Test D, (**c**) Test E, (**d**) Test F.

Test C produced an average thickness of 95.13 μm and a maximum thickness of 172.1 μm (Figure 6a). The focus variation measurements for Test D (1.2 J) showed an average thickness of 39.82 μm, with the smallest thickness measured at the edges (average of 8.06 μm), and patches of bonding of different coating thicknesses are apparent in the colour scale bar in Figure 6b. The 3D scan of Test F (0.3 J) indicated that the coating was applied uniformly across most of the PCBN, with an average thickness of 53.73 μm, however coating was not present at one of the edges and it was about 66.17 μm in another edge (Figure 6d). Combined optical microscopic analyses and focus variation measurements for Test E suggested that the coating was not applied uniformly, that the average thickness was 21.30 μm, and that the maximum coating thickness was 64.19 μm in the centre of the sample (Figure 6c). This larger deposition of coating near the centre of the sample may be due to the low weld speed adopted (0.01 m·s^−1^) and the sonotrode being in contact with the centre of the sample for an extended bonding time (1 s), therefore promoting an accumulation of CNT powder in the centre of the specimen.

### 3.2. Effect of Weld Speed, Force, and Amplitude on Micro-Hardness and Reliability of Coating

**Figure 7:**
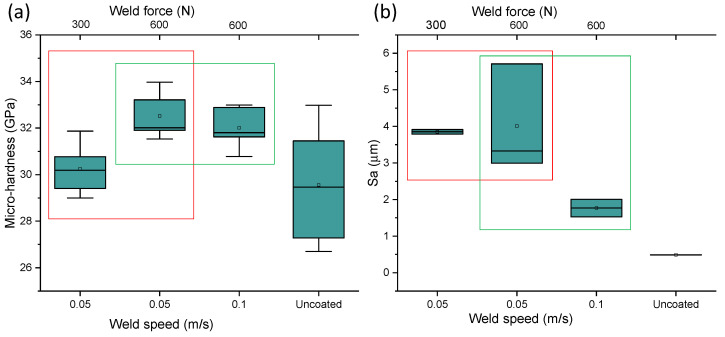
(**a**) Boxplot of the variation of micro-hardness as a function of weld force and weld speed, (**b**) boxplot of the effect of weld speed and weld force on the 3D surface roughness coating variation.

Weld speed was varied to investigate its impact on the hardness of the coated samples. An optimisation window for bonding parameters was disclosed, using a maximum welding force (600 N) and doubling the speed seems to reduce the hardness by 2% (green window in Figure 7a), although in both cases, the average micro-hardness values are 8–10% above the uncoated materials. This result is in accordance with investigations by Kong et al. [23], in which it was reported that a decrease of weld speed would favour strain hardening due to the increased energy in the bonding area (at parity of pressure and amplitude). At speed of 0.05 m·s^−1^, there seems to be an optimal force needed to achieve hardness above the benchmark (red window in Figure 7a). Bonding at half of the force (at equal speed) lessened the scatter between first and third quartiles (red window in Figure 7b) of the 3D Sa values. However, doubling the speed (at equal load) promoted a reduction of average Sa by 100% (green window in Figure 7b). A good compromise between micro-hardness and good surface integrity (Sa) was achieved for Test A (speed, 0.1 m·s^−1^; force 600 N).

**Figure 8:**
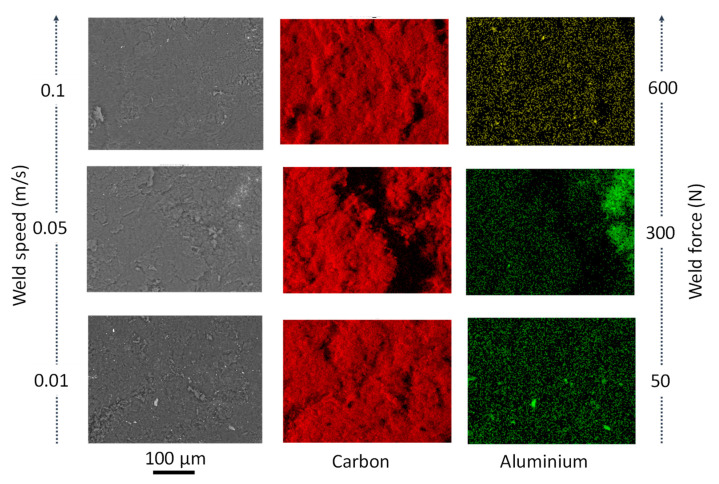
Schematic of the effect of weld speed and weld force variation on the elemental composition results for PcBN samples post-coating (from top to bottom, Tests A, C, and B).

From the topographical analysis, the largest surface roughness was identified when coating using the largest weld speed (0.1 m·s^−1^), however a strong correlation between thickness and speed was found. Coating at testing condition A (0.1 m·s^−1^) resulted in an average thickness of 86.45 μm with maximum deposition in the centre of the sample (135.34 μm). Reducing the speed (0.05 m·s^−1^ in Test C) increased the average thickness up to 95.13 μm, diminishing the homogeneity of the coating, which appeared porous in extended areas of the sample (Figure 8). A further reduction of speed (0.01 m·s^−1^ in Test B) in combination with a reduced bonding pressure (5 MPa) and force achieved a coating with an average thickness of 47.09 μm.

**Figure 9:**
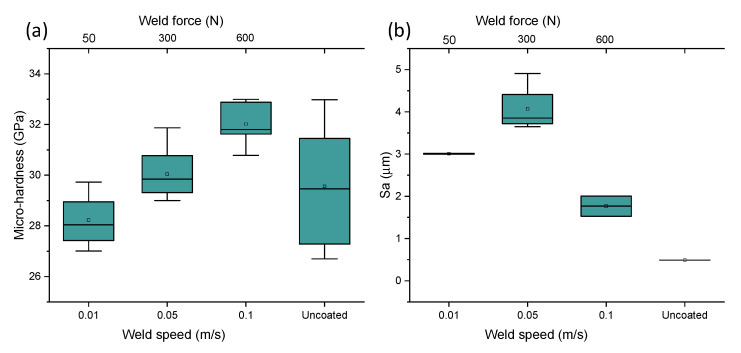
(**a**) Boxplot of the variation of micro-hardness as a function of weld force and weld speed, (**b**) boxplot of the effect of weld speed and weld force on the 3D surface roughness coating variation.

A clear trend of increased micro-hardness was revealed by the indentations and an optimisation window for bonding parameters was identified. A simultaneous increase of weld speed and force promoted a drop in Sa values and an improvement of micro-hardness between 8% and 10% (Figure 9a), however a threshold of parameters exists (speed < 0.05 m·s^−1^, force < 50 N) below which strain softening occurs (micro-hardness reduced by 2% compared to benchmark). O’Brien [24] showed that the temperature rise in ultrasonic bonding process can reduce stresses and promote atomic diffusion and recrystallisation, however an increase of processing temperature is responsible for reducing strain hardening.

The impact of oscillation amplitude was investigated in relation to density of bonded area, thickness of the coating, and micro-hardness. In this study, for all coating conditions, the amplitude was kept in the range 12–21 μm (Table 2) with a maximum of 21 μm for Tests A, D, and G, a minimum of 12 μm for Tests B and C, and 16 μm for Tests E and F. For Test B, the low amplitude and low bonding pressures (5 MPa) were insufficient to generate enough frictional heat, therefore the MWCNTs did not bond to the substrate. This is in accordance with findings from Jones and Powers [25], who demonstrated that oscillation of amplitude determines the amount of elastic/plastic deformation at the bonding interface, later also validated by Friel et al. [26], who demonstrated that high bond energy results in better bond strength and greater plastic flow.

Data analysis revealed a clear trend between amplitude and hardness. A small change in hardness was detected when changing the amplitude only, hence the amplitude has a negligible effect on the hardness unless accompanied by the effect of bonding pressures. At an amplitude of 21 μm, the hardness was increased only for bonding at pressures in the vicinity of 60 MPa (conditions A and G), while at a pressure of 30 MPa, the coating hardness was comparable to the uncoated sample (condition D). This can be explained considering the achievable temperatures in ultrasonic bonding. Previous studies proved that there is a direct link between amplitude and measured temperature. For amplitudes of oscillation above 25 μm, the peak temperature was measured under 523 K, and for amplitudes of oscillation below 25 μm, the temperature was reported to be less than 373 K [8,16,17]. Therefore, coating just below the mentioned threshold (21 versus 25 μm), however at maximum bonding pressure, would have favoured temperatures in the range 400–523 K, well above the deformation temperature for 90% cBN crystals (473 K), causing plastic deformation of the cBN grains, strain hardening, and therefore, providing a strengthening effect at the bonding interface.

### 3.3. Characterisation of MWCNTs Ante/Post-Coating Process

**Figure 10:**
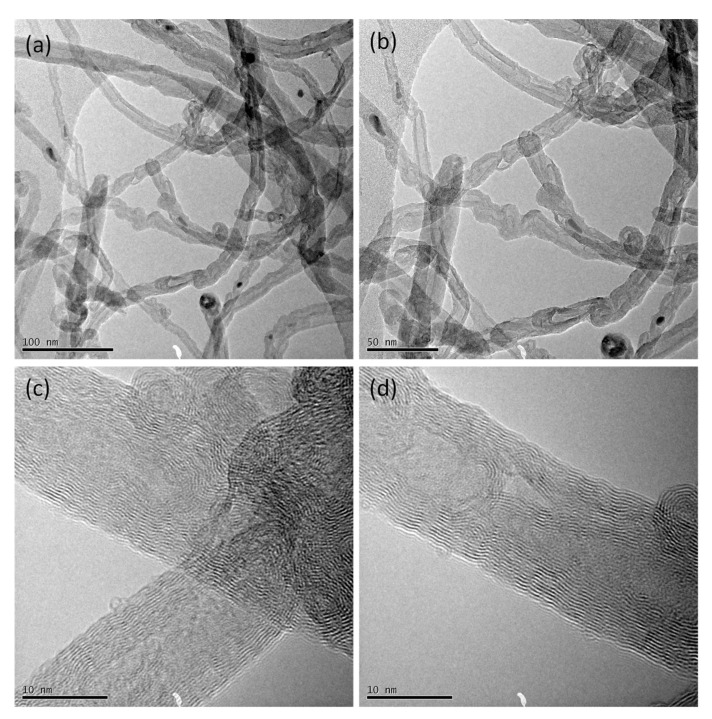
Transmission electron microscopy (TEM) of multi-walled carbon nanotubes (CNTs) grown via the catalytic carbon vapour deposition (CCVD) process at: (**a**) 100 nm scale bar, (**b**) 50 nm scale bar, (**c**,**d**) 10 nm scale bar.

Transmission electron microscopy was used to characterize the as-received CNTs prior to the coating process, and results of the characterisation are shown in Figure 10, which confirmed that the CNTs were multi-walled (Figure 10c,d) with diameters between 10 and 20 nm and lengths between 10 and 30 μm (Figure 10a,b).

**Figure 11:**
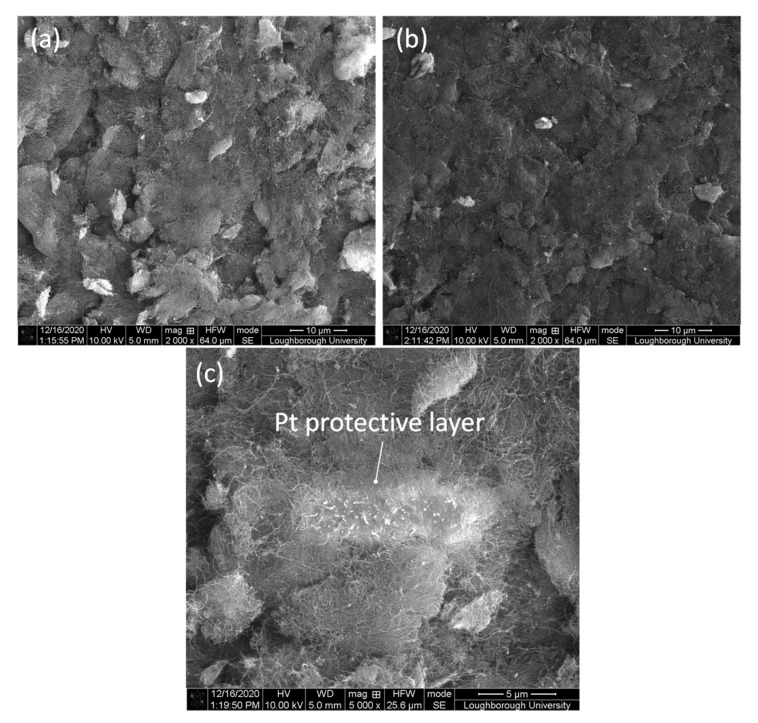
SEM images of the PCBN samples coated with CNTs: (**a**) condition A, (**b**) condition G, (**c**) higher resolution image of platinum coating for condition A. All images were taken in top view.

FIB-SEM analyses were conducted for coating conditions A and G, which showed the highest micro-hardness values. The images achieved from SEM analyses are shown in Figure 11 for coating condition A (Figure 11a) and coating condition G (Figure 11b). After SEM analyses of these two coating conditions, the samples were prepared for focus ion beam milling. The application of a platinum layer (length 10 μm, width 3 μm, and thickness 1 μm) was carried out in the site of milling in order to protect the entire exposed surface prior to FIB milling, as shown in Figure 11c for coating condition A.

**Figure 12:**
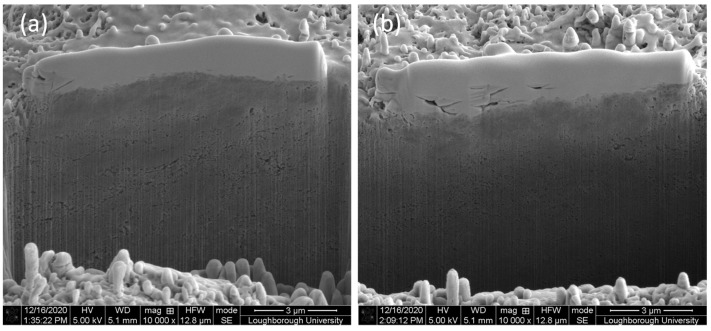
SEM images showing a slice milled through the CNT coating: (**a**) condition A, (**b**) condition G. All images were taken with a sample tilt of 50°.

The results from FIB milling are depicted in Figure 12 for coating condition A (Figure 12a) and coating condition G (Figure 12b). Differences between the thickness of the platinum layer among the two prepared FIB cuts can be observed in Figure 12. In particular, the sample coated at condition G revealed some porosity in the preparation of the FIB area and a variable thickness across the length of the Pt protective layer. This is due to the difference in 2D and 3D surface roughness between coated samples A and G, with Sa values for coating G twice larger than coating A (boxplots in Figure 2d).

**Figure 13:**
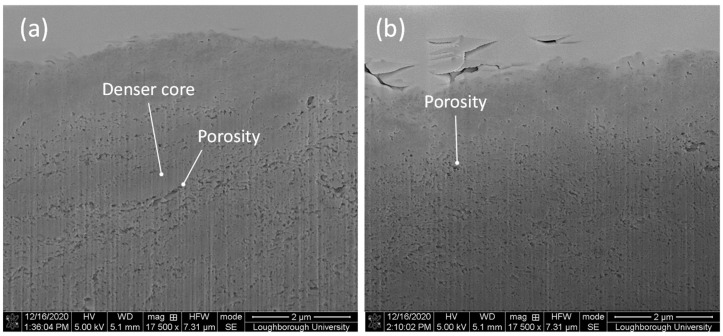
SEM high-resolution images showing a slice milled through the CNT coating: (**a**) denser core and elongated porosity in condition A, (**b**) circular porosity in condition G. All images were taken with a sample tilt of 50°.

Although the same pressure was exerted in conditions A and G, the high-resolution SEM images of the FIB cross-section (at a depth of 1 μm from the FIB coating) revealed different densities for CNTs post-coating process. A denser structure with patches of elongated porosity was achieved at bonding condition A (Figure 13a) and circular porosity was revealed at bonding condition G (Figure 13b). The microstructure achieved in condition A resembles the core-sheath structure achieved by milling CNT yarns with spun twist density of 25 mm^−1^ [27], while the one in condition B, the core-sheath structure, was achieved for spun twist density of 20 mm^−1^ [27]. Although the bonding pressure exerted in conditions A and G is the same, condition A was achieved at faster speed and double power in half weld time, causing larger bonds between the CNTs and elongated dense patches, with areas of 250 nm by 1.5 μm (Figure 13a). Bonding at condition G occurred slower, in a longer weld time, and half of the power than in condition A, decreasing exposure in the instantaneous contact area between MWCNTs and sonotrode. Although the mechanism for bonding is not fully understood, the visual analyses of bonding’s cross-sections between CNTs (Figure 13) is in line with the hypothesis of plastic flow caused by the weld pressure for conditions A and G, substantiated by the highest increase of hardness.

## 4. Conclusions

This paper investigated the use of ultrasonic consolidation for applying thick coatings (20–100 μm) on ultra-hard PcBN materials. We reported for the first time a window of operating conditions for the MWCNTs to bond to the dissimilar substrate. For five coating conditions, micro-hardness values of the coating were in the range of the uncoated PcBN values, however uniformity of mechanical properties post-process was revealed by the smaller scatters in the boxplot of micro-hardness values. For two coating conditions, it was seen that bonding pressure has a higher impact than energy on achieving hardness values above 32 GPa, higher than the average value in the uncoated sample (29 GPa). The combined effect of bonding forces (600 N) and bonding pressures (60 MPa) favoured frictional heat generation during the bonding process, promoting a rise in temperature at the boundary due to vibration and atoms bonding, favouring plastic deformation in 90% cBN material for temperatures above 473 K cBN. The simultaneous increase of weld speed and force promoted a drop in Sa values and an improvement of micro-hardness between 8% and 10%, however a threshold of parameters was revealed (speed < 0.05 m·s^−1^, force < 50 N), below which strain softening occurred (micro-hardness reduced by 2% compared to benchmark). The investigations revealed that the amplitude had a negligible effect on the hardness unless accompanied by the effect of bonding pressures: low amplitudes and low bonding pressures (5 MPa) were insufficient to generate enough frictional heat, therefore the MWCNTs did not bond to the substrate. FIB milling analyses revealed a denser structure with patches of elongated porosity for bonding condition A and circular porosity for bonding condition G. Interestingly, the bonding between MWCNTs achieved using ultrasonic consolidation resembles the core-sheath structure achieved in the manufacture of CNT yarns with spun twist density in the range 20–25 mm^−1^.

This study investigated the unconventional application of ultrasonic consolidation to coat PcBN materials with CNTs, achieving bonding between carbon nanotubes comparable to the core-sheath structure attained in the manufacture of CNT yarns. The analysis of the complete set of results showed that the full range of parameters should be considered to establish the most suitable bonding mechanism for the dissimilar materials. The proposed method paves the way for the novel design of functional coatings with attunable properties (i.e., thickness and hardness) and therefore, improved productivity in the machining of aerospace and automotive parts.

## Figures and Tables

**Table 1:** Parameter values at each level used for PcBN design of experiment (DOE).

Parameter	Code	Level 1	Level 2	Level 3
Welding force (N)	A	50	300	600
Weld speed (m·s^−1^)	B	0.01	0.05	0.1
Weld power (W)	C	0.5	3	6
Weld time (s)	D	0.1	0.2	1

**Table 2:** DOE of bonding parameters used for coating the PcBN samples.

Test	Amplitude (μm)	Force (N)	Weld Speed (m·s^−1^)	Weld Time (s)	Power (W)	Weld Energy (J)	Bonding Pressure (MPa)
A	21	600	0.1	0.1	6	0.6	60
B	12	50	0.01	1	0.5	0.5	5
C	12	300	0.05	0.2	3	0.6	30
D	21	300	0.05	0.2	6	1.2	30
E	16	300	0.01	1	3	3	30
F	16	300	0.1	0.1	3	0.3	30
G	21	600	0.05	0.2	3	0.6	60

**Table 3:** Analysis of variance (ANOVA) results on coating micro-hardness of ultrasonic bonding experiments.

Source	Sum of Squares	Mean Square	Degrees of Freedom (DF)	F Ratio	*p*-Value
D E F	5.30	2.65	2	2.60	0.103
B A	35.79	35.79	1	34.19	3.84 × 10^−4^
C A	7.79	7.79	1	7.259	2.73 × 10^−2^
E A	25.50	25.50	1	23.05	1.35 × 10^−3^
F A	10.92	10.92	1	12.07	8.38 × 10^−3^
G B	46.91	23.45	1	19.70	0.001
G C	12.95	12.95	1	11.05	0.010
G D	12.95	12.95	1	11.05	0.010
G E	34.29	34.29	1	28.50	6.95 × 10^−4^
G F	16.90	16.90	1	16.88	0.003

**Table 4:** ANOVA results on coating thickness of ultrasonic bonding experiments.

Source	Sum of Squares	Mean Square	DF	F Ratio	*p*-Value
D E F	2938.03	1469.01	2	3.55	0.051
E A	10,096.51	10,096.51	1	13.59	0.006
CE	13,042.93	13,042.93	1	9.67	0.014
GD	12,008.30	12,008.3	1	22.87	0.001
GE	19,942.48	19,942.48	1	27.91	7.43 × 10^−4^

## Data Availability

Not reported any data.
